# Supplementary material for: Investigating slant curves within Lorentzian doubly warped product manifolds
Source: PLoS One. 2025 Apr 30;20(4):e0320678. doi: 10.1371/journal.pone.0320678 (PMC12043243; doi:10.1371/journal.pone.0320678)
Supplement: Attachment — Submitted filename: PONE_D_24_52708_review.pdf [file pone.0320678.s001.pdf]

Summary

The authors extend a previous classification of slant curves in Minkowski space to the family of Robertson-Walker (RW) metrics, giving curvature and torsion formulae for timelike, spacelike, and null curves. Their approach emphasizes realizing the generic (1,2)-RW metric as (1,2)-Minkwoski space endowed with a doubly-warped product structure.

Major/minor comments

The work is thorough and largely very well-written – I wish to commend the authors on their clear writing and accessible presentation of the material. The scope of the work is limited, and the authors tell its story rigorously and effectively without sacrificing clarity. While I do have some suggestions to improve the paper, I think this work is strong overall and suitable for publication after revision.

Exposition/Discussion/Intepretation

The manuscript's work is well-motivated by a clear and compelling introduction and, generally speaking, exposition is well-written and helpful when it is present. However, sections 3 and 4 are more focused on presenting calculations than helping the reader contextualize their motivation, interpret their meaning, or appreciate their implications. This would be fine (if still a bit difficult to parse) in the presence of a discussion section (or a longer, more interpretive conclusion), but as it stands feels in need of narrative through-lines. I would find this paper much more digestible if it padded its technical results with more explanation and/or interpretation of those results. Relatedly, the addition of minimal nontrivial examples of important results and/or definitions would be extremely illustrative and helpful.

Frenet-Serret equations

I found section 2.3 somewhat confusing in its treatment of timelike, spacelike, and null curves; this is a crucial section of the paper, and I think it requires both more detail and a more structured organization. In particular, comparing and contrasting minimal nontrivial examples of timelike, spacelike, and null Frenet apparatus would be illuminating. Unfortunately, after reading this section, I am left fairly unclear on ***why*** the Frenet-Serret equations should take the form of equations (2.6*) and (2.7*) in the spacelike and null curve cases, or what significance/intuition I should attach to them. Here is a brief list of the questions I was felt confused by/unable to resolve while reading this section:

(a) "If the covariant derivative of the tangent vector along a curve is spacelike, the curve is said to be a null curve. [...] is called a unit speed curve, and it lies on a null curve α." – why would this be a lightlike curve and not a spacelike one? If it is a null curve, how does a spacelike covariant derivative relate to the vanishing norm of the tangent vector?

(b) Why does the paragraph beginning "A non-null curve $\alpha(s)$ [...]" not instead say "A timelike curve $\alpha(s)$ [...]"? This definition is only used in the timelike case later. (Also, I think it may be clearer to swap this paragraph with the previous one.)

(c) Why is it a desired property that the norm of the covariant derivative of a Frenet curve be identically 0 if that curve is spacelike and nonvanishing if that curve is timelike?

(d) In equations (2.6a-d), is it still the case that $T = \alpha'(s)$? If not, what is the relationship between $\alpha(s)$ and the rest of the Frenet apparatus?

Lastly, this is a smaller issue, but I would also recommend writing out the Frenet curve definition as its own definition (e.g., Defintion 2.3), or at least typographically offsetting it in some way that makes it easier to refer back to.

Style

This is mostly a cosmetic complaint, but it would also help with clarity: please state common theorem data before enumerating cases in Theorems 3.1 and 3.2. (e.g., "Let $\alpha(s)$ be a unit-speed slant Frenet curve. (a) if α timelike, then ... (b) if α spacelike, then ... (c) if α null, then ... etc). Additionally, please state that $\gamma \in C^\infty(J)$ somewhere in the theorem statement.

Finally, Corollaries 4.1 and 5.1 would be much more readable if separated into individual corollaries.

Line items

1. In the last paragraph of the introduction, the acronym "GRW" is used without definition, and I don't know what it means.
2. Equation (2.1a) should read $g(\nabla_{\partial_t}\partial_t, \partial_t)$, and it might be worth explicitly mentioning that this vanishes specifically because h does not depend on t (per Lemma 3.1 of Du and Wang's cited paper)
3. The ϵ_i in equations (2.5a-e) are labeled as "casual characters" and I think should be "causal characters."
4. In the first paragraph of section 3, please provide citations illustrating that "slant curves have signifcant implications in fields such as computer graphics, robotics, and biophysics."
5. In the line following equation 3.5, "Form" should be "From" and I'm cpnfused as to why $h = h(\alpha_1(s))$ would hold instead of $h = h(\alpha_2(s), \alpha_3(s))$.
6. In the first line of the proof of Proposition 3.5, $g(T, \partial_t, T)$ is written (which of course is not an expression that makes sense), so it's unclear why the first term of equation (3.17) is ηT.
7. A few times in sections 3 and 4, the phrase "for some $\alpha \in C^\infty(J)$ shows up – it seems like α should be γ in these lines, but it would be easier to be certain if data were clearly defined in the statement of theorems and propositions.
