# Supplementary material for: Investigating slant curves within Lorentzian doubly warped product manifolds
Source: PLoS One. 2025 Apr 30;20(4):e0320678. doi: 10.1371/journal.pone.0320678 (PMC12043243; doi:10.1371/journal.pone.0320678)
Supplement: Attachment — Submitted filename: Refree reply.pdf [file pone.0320678.s002.pdf]

Dear Editor,

I would like to express my sincere gratitude for the time and effort you have dedicated to revising our manuscript. We truly appreciate your thorough examination of every detail in the study to ensure its accuracy and readiness for publication in *Plos one*. Your careful attention to each aspect of the manuscript has been invaluable in improving the overall quality of our work.

In addition, we would like to thank you for your insightful suggestions, which have helped us refine our analysis and strengthen the clarity of the paper. We are confident that the revisions made in response to your feedback, as well as the feedback from the reviewers, have significantly enhanced the manuscript.

As requested, we have carefully addressed each referee's comment in detail. Please find attached the response letter, which outlines how we have incorporated their suggestions and made the necessary revisions.

Reviewer 1

We have carefully considered each of your comments and have provided a detailed response to ensure that all necessary revisions have been implemented effectively:

Comment 1.
Page 2, paragraph 4, please state the full name of acronym GRW.

Response: We defined the acronym as **Generalized Robertson–Walker (GRW).**

Comment 2.
Page 6, Proposition 3.1, please capitalized the beginning word "if" in point 1 and 2.

Response: We made the required.

Reviewer 2

We sincerely appreciate your dedicated time and effort in reviewing our manuscript. Your insightful comments and precise observations have greatly improved our work's quality and clarity. It is evident that you are deeply engaged with the latest developments in this field, and we are grateful for your thorough and thoughtful assessment.

Your feedback has helped us refine key aspects of our study, and we have carefully addressed each of your suggestions in the detailed responses below. We believe that these revisions have strengthened the manuscript significantly, and we truly value your expertise in guiding us toward a clearer and more robust presentation of our findings.

Please find below our point-by-point responses to your comments.

Part I. Frenet-Serret equations

Comment 1.
why the Frenet-Serret equations should take the form of equations (2.6*) and (2.7*) in the spacelike and null curve cases,

Response
We added the following reference:

Walrave, Johan. Curves and Surfaces in Minkowski Space. PhD diss., Doctoral thesis, KU Leuven, Faculty of Science, Leuven, 1995.

This work discusses in detail how to derive the Frenet-Serret equations in all cases.

Comment 2.
Why would this be a lightlike curve and not a spacelike one? If it is a null curve, how does a spacelike covariant derivative relate to the vanishing norm of the tangent vector?

Response
If the tangent vector α' were spacelike, then $g(\alpha', \alpha') > 0$, which contradicts the assumption that the curve is null. The spacelike nature of $\nabla_{\alpha'}\alpha'$ does not affect the fact that α' is always lightlike; it merely indicates that the curve is not a geodesic and that there is some nontrivial acceleration in a spacelike direction.

A curve is classified as null based on its tangent vector α', not its acceleration. The fact that $\nabla_{\alpha'}\alpha'$ is spacelike means the curve is accelerating in a spacelike direction, but the curve itself remains lightlike because its tangent vector is always null.

Comment 3.
Why does the paragraph beginning " A non-null curve $\alpha(s)$ [...]" not instead say " A timelike curve $\alpha(s)$ [...]" ? This definition is only used in the timelike case later. (Also, I think it may be clearer to swap this paragraph with the previous one.)

Response
When discussing non-null curves in Minkowski space, we classify them into three types based on the causal nature of the tangent, normal, and binormal vectors:

1. (Spacelike, Spacelike, Timelike)
2. (Timelike, Spacelike, Spacelike)
3. (Spacelike, Timelike, Spacelike)

These classifications provide a generalized framework for studying non-null curves. Depending on the specific study, we select the appropriate type based on the properties required for analysis.

Comment 4.
Why is it a desired property that the norm of the covariant derivative of a Frenet curve be identically 0 if that curve is spacelike and nonvanishing if that curve is timelike?

Response
The desired property, based on the norm of $\nabla_{\alpha'}\alpha'$, can be summarized in the following cases:

**Curve Type**	**Norm of $\nabla_{\alpha'}\alpha'$**	**Interpretation**
Spacelike Frenet Curve	Zero (Spacelike)	Represents a geodesic (straightest path in a spacelike direction).
Spacelike Frenet Curve	Non-Zero (Timelike or Spacelike)	Represents a non-geodesic motion with curvature.
Timelike Frenet Curve	Zero (Timelike	Represents a timelike geodesic (free-fall motion).
Timelike Frenet Curve	Non-Zero (Spacelike)	Represents an accelerating massive particle (non-geodesic motion).

Note: The appropriate case is chosen based on the specific focus of our study in this part of the manuscript.

Comment 5.
In equations (2.6a-d), is it still the case that $T = \alpha'(s)$? If not, what is the relationship between $\alpha(s)$ and the rest of the Frenet apparatus?

Response
It still holds that $T = \alpha'(s)$ as the tangent vector.

Comment 6.
Lastly, this is a smaller issue, but I would also recommend writing out the Frenet curve definition as its own definition (e.g., Defintion 2.3), or at least typographically offsetting it in some way that makes it easier to refer back to.

Response
We added the definition of the Frenet curve at the beginning of Section 2.3, *Frenet-Serret Equations*.

<u>Part II. Style</u>

Comment 1.
This is mostly a cosmetic complaint, but it would also help with clarity: please state common theorem data before enumerating cases in Theorems 3.1 and 3.2. (e.g., ”Let $\alpha(s)$

be a unit-speed slant Frenet curve. (a) if $\alpha(s)$ timelike, then… (b) if $\alpha(s)$ spacelike, then ... (c) if $\alpha(s)$ null, then ... etc)

Response
We stated a common theorem, making the presentation more scientific and clearer.

Comment 2.
Additionally, please state that $\gamma \in C^{\infty}(J)$ somewhere in the theorem statement.

Response
We added it at the end of the theorem's statement.

Comment 3.
Finally, Corollaries 4.1 and 5.1 would be much more readable if separated into individual corollaries.

Response
We separated them into three separate corollaries.

Part III. Line items

Comment 1.
In the last paragraph of the introduction, the acronym ”GRW” is used without definition, and I don’t know what it means.

Response
We defined the acronym as **Generalized Robertson–Walker (GRW).**

Comment 2.
Equation (2.1a) should read $g\left(\nabla_{\partial_t}\partial_t, \partial_t\right)$, and it might be worth explicitly mentioning that this vanishes specifically because h does not depend on t (per Lemma 3.1 of Du and Wang’s cited paper)

Response
There was a typo, and we have fixed it to be like $g\left(\nabla_{\partial_t}\partial_t, \partial_t\right)$.

Comment 3.
The ϵ_i equations (2.5a-e) are labeled as ”casual characters” and I think should be ”causal characters.”

Response
There was a typo, and we have fixed it to be “causal characters”.

Comment 4.
In the first paragraph of section 3, please provide citations illustrating that ”slant curves have signifcant implications in fields such as computer graphics, robotics, and biophysics.”

Response
We have added a reference that highlights the significant implications of slant curves in the study of these fields.

Comment 5.
In the line following equation 3.5, ”Form” should be ”From” and I’m confused as to why $h(\alpha_1)$ would hold instead of $h\big(\alpha_2(s), \alpha_3(s)\big)$

Response
There was a typo, and we have fixed it as h is defined as $h\big(\alpha_2(s), \alpha_3(s)\big)$.

Comment 6.
In the first line of the proof of Proposition 3.5, $g(T, \partial_t, T)$ is written (which of course is not an expression that makes sense), so it’s unclear why the first term of equation (3.17) is ηT.

Response
There was a typo, and we have fixed it as $g(T, \partial_t)$.
The first term of Equation (3.17) is ηT. It follows directly from Equation (3.5) after differentiating the curve $\alpha(s)$, taking the dot product of $\alpha'(s)$with ∂_t according to the defined metric, and setting $H^{*\prime} = 1$ for simplicity. This yields the desired outcome.

Comment 7.
A few times in sections 3 and 4, the phrase ”for some $\alpha \in C^\infty(J)$ shows up – it seems like α should be γ in these lines, but it would be easier to be certain if data were clearly defined in the statement of theorems and propositions.

Response
There were typos, and we have fixed them. We also revised them in these two sections.

Once again, we sincerely thank you for your time and effort in reviewing our manuscript. Your precise and insightful comments have significantly improved the quality and clarity of our work. We truly appreciate your valuable contributions to this revision.

Sincerely,
The authors
